# Stability of Tin- versus Lead-Halide Perovskites: Ab Initio Molecular Dynamics Simulations of Perovskite/Water Interfaces

**DOI:** 10.1021/acs.jpclett.2c00273

**Published:** 2022-03-04

**Authors:** Waldemar Kaiser, Damiano Ricciarelli, Edoardo Mosconi, Asma A. Alothman, Francesco Ambrosio, Filippo De Angelis

**Affiliations:** † Computational Laboratory for Hybrid/Organic Photovoltaics (CLHYO), Istituto CNR di Scienze e Tecnologie Chimiche “Giulio Natta” (CNR-SCITEC), Via Elce di Sotto 8, 06123 Perugia, Italy; ‡ Department of Chemistry, Biology and Biotechnology, University of Perugia, Via Elce di Sotto 8, 06123 Perugia, Italy; § Chemistry Department, College of Science, King Saud University, Riyadh 11451, Kingdom of Saudi Arabia; ∥ Department of Chemistry and Biology “A. Zambelli”, University of Salerno, Via Giovanni Paolo II 132, 84084 Fisciano, Salerno, Italy; ⊥ CNST@Polimi, Istituto Italiano di Tecnologia, Via Pascoli 70/3, 20133 Milano, Italy

## Abstract

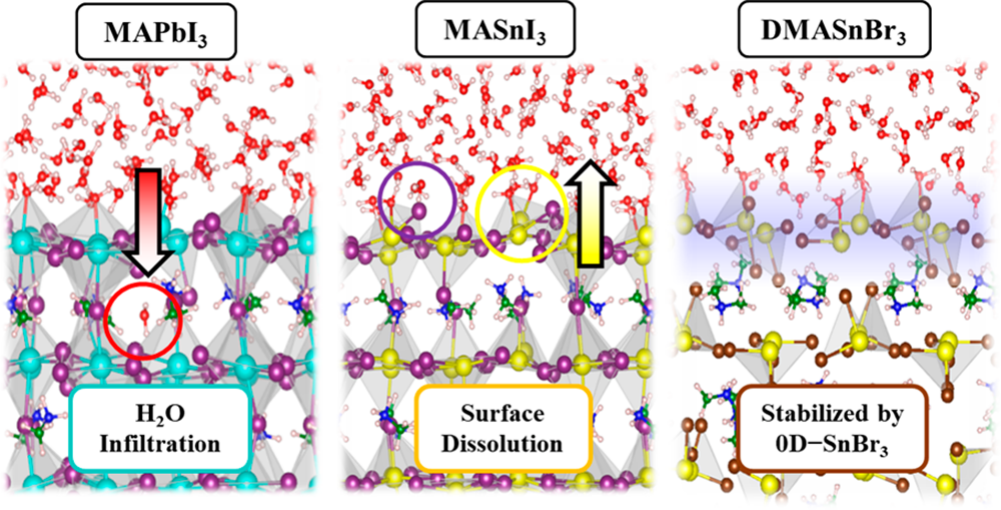

Tin-halide perovskites (THPs) have emerged as promising lead-free perovskites for photovoltaics and photocatalysis applications but still fall short in terms of stability and efficiency with respect to their lead-based counterpart. A detailed understanding of the degradation mechanism of THPs in a water environment is missing. This Letter presents *ab initio* molecular dynamics (AIMD) simulations to unravel atomistic details of THP/water interfaces comparing methylammonium tin iodide, MASnI_3_, with the lead-based MAPbI_3_. Our results reveal facile solvation of surface tin–iodine bonds in MASnI_3_, while MAPbI_3_ remains more robust to degradation despite a larger amount of adsorbed water molecules. Additional AIMD simulations on dimethylammonium tin bromide, DMASnBr_3_, investigate the origins of their unprecedented water stability. Our results indicate the presence of amorphous surface layers of hydrated zero-dimensional SnBr_3_ complexes which may protect the inner structure from degradation and explain their success as photocatalysts. We believe that the atomistic details of the mechanisms affecting THP (in-)stability may inspire new strategies to stabilize THPs.

Metal halide perovskites belong to the most promising semiconducting materials for energy conversion from renewable sources in the form of photovoltaics^1^ and photocatalysis^2^ thanks to their favorable optoelectronic properties, such as direct and tunable band gaps,^3^ fast charge transport,^4^ low nonradiative recombination,^5^ and low-temperature processing capabilities from solution.^6^ Thin film solar cells based on lead-halide perovskites (LHPs) have recently reached 25.2% efficiency with significant long-term stability,^7^ rivaling silicon solar cells.

A tremendous drawback which may limit the industrial market potential of state-of-the art perovskite photovoltaics is the presence of lead in the B-site of the ABX_3_ perovskite crystal structure. Recent reports^8,9^ have underlined the detrimental impact of lead on the environment and human health, and have further raised the need for lead-free alternatives. Tin-halide perovskites (THPs) have emerged as one of the most promising lead-free alternative for photovoltaics^10−12^ with record efficiencies of 14.6%^13^ in pure THPs and beyond 20% in mixed tin–lead perovskites.^14−17^ Despite having an optimal band gap (∼1.2–1.3 eV) for photovoltaic applications, THP performance and stability are still behind their lead-based counterpart. One of the critical aspects limiting the performance and the stability of THPs is the facile oxidation of Sn(II) to Sn(IV),^18,19^ which may induce a large self-p-doping in the perovskite bulk and deep electron trap states at the perovskite surface.^20−22^ Experimental reports demonstrate THPs degradation even in well-encapsulated thin films under exposure of light or under elevated temperatures.^23−25^

Understanding of the perovskite (in-)stability and associated degradation mechanisms is of utmost importance to achieve reliable and long-term stable THPs. Previous studies have shed light on the degradation of the lead-based counterpart, methylammonium lead iodide, MAPbI_3_. *Ab initio* molecular dynamics (AIMD) simulations^26^ were reported on the degradation of MAI-terminated surfaces by a rapid solvation process which occurs by the bonding of water molecules to the Pb atoms with subsequent release of surface iodine atoms. Metadynamics simulations showed that, after the initial Pb–I bond breaking, further hydration of iodine provides the required energy to overcome the I^–^-MA^+^ attraction.^27^ On the contrary, the PbI-terminated surface is more robust to degradation, while water molecules may enter into the MAPbI_3_ bulk and form intermediate hydrated phases,^26,28,29^ which is a key step of perovskite degradation upon water exposure.^30−32^ Water-intercalated MAPbI_3_ readily forms PbI_2_ vacancy complexes and induces electronic trap states.^33^ The presence of water can be both detrimental or beneficial to the excited state lifetime, depending on the level of humidity exposure.^34^ Light exposure can further drive perovskite degradation by dissociating water molecules to OH^–^, which may deprotonate the MA cation followed by desorption of CH_3_NH_2_.^35−37^ Interestingly, partial replacement of iodine with bromine can enhance perovskite stability toward water and oxygen.^37,38^

Recent studies connected the degradation of THPs with the release of SnO_2_ and SnI_4_, while in mixed tin-lead perovskites I_2_ formation also was observed.^24^ The presence of oxygen and moisture further accelerates THP degradation by formation of SnI_4_.^39,40^ Density functional theory (DFT) simulations suggest that adsorbed water or oxygen, by formation of H–I or Sn–O bonds, strongly affects the Sn–I bonds at the methylammonium tin iodide, MASnI_3_, surface,^39,41^ which is less significant in the case of MAPbI_3_.^41^ The presence of water molecules in THPs may further reduce light absorption and lead to faster charge relaxation.^42^ Several strategies such as metal doping,^43^ Lewis-base post-treatment,^44^ or capping 3D THP with analogue 2D large-cation perovskites^45^ were proposed to mitigate tin oxidation, which in turn may stabilize THPs.

Despite recent efforts, a clear mechanistic picture of the factors affecting THP stability in a water environment is still missing. Here we report comparative AIMD simulations of MASnI_3_ with the prototypical lead-based MAPbI_3_ perovskite, Figure 1a,b, thus assessing the impact of the B-site metal on the interaction with water. A comparison with dimethylammonium tin bromide, DMASnBr_3_, Figure 1c, a water-stable perovskite catalyst,^46^ is also reported, which allows us to comparatively evaluate the concomitant impact of the A-site and X-site variation on the perovskite/water interface stability. Note that AIMD results on the water-induced degradation of MAPbI_3_ are partially reproduced from our previous analysis,^26^ together with additional statistical analysis, to provide a direct comparison with the prototypical MAPbI_3_/water interface as representative of the lead-based counterpart. Our results reveal a dissolution of the SnI_2_-terminated MASnI_3_ surface under water which is initiated by the formation of Sn–O bonds and the subsequent breaking of axial Sn–I bonds. The MAPbI_3_ surface, despite showing an increased amount of adsorbed water molecules, preserves its inorganic Pb–I framework without a substantial difference in the radial pair distribution between bulk and surface. However, water infiltration is observed, which presents the first step of MAPbI_3_ degradation. Our results indicate that the high water stability of DMASnBr_3_ is due to the presence of an amorphous surface layer made of disconnected, zero-dimensional SnBr_3_ complexes, stabilized by strong Sn–Br bonds, which strongly bind water molecules and protect the DMASnBr_3_ bulk.

**Figure 1:**
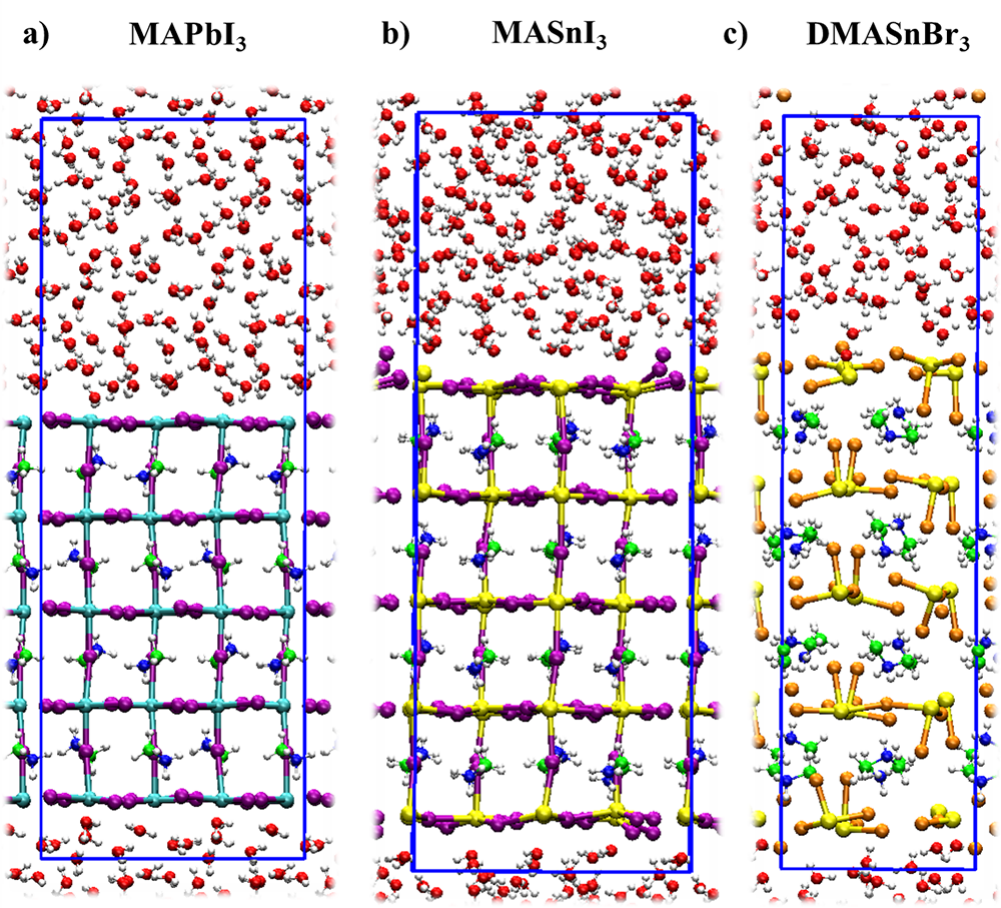
Structural model of the (a) MAPbI_3_, (b) MASnI_3_, and (c) DMASnBr_3_ perovskite/water interfaces. The colors of the atoms are cyan: Pb, yellow: Sn, purple: I, orange: Br, green: C, white: H, blue: N, red: O. Further details on the structural models are provided in the Computational Details.

We start our analysis by comparing the interaction of the (001) MI_2_-terminated (M = Pb, Sn) tetragonal MAPbI_3_ and MASnI_3_ perovskite surfaces with the surrounding liquid water environment (see Computational Details for model setup and AIMD simulation details). The (001) surface belongs to the most stable perovskite surfaces^47^ and has been subject to several studies on the electronic properties of lead-^26,28,32^ and tin-based perovksites.^21,22,39,45^ The radial pair distribution function g^M–O^(r) (M = Sn, Pb), averaged over the AIMD trajectories, indicates a strong interaction of the Sn surface atoms with the water oxygen atoms, as shown by the peak located at 2.43 Å; see Figure 2a. The g^Pb–O^(r) of the MAPbI_3_ surface comparatively shows a substantially broadened peak centered at around 2.53 Å. The interaction at the perovskite/water interface mainly occurs via the O lone pair electrons and the M^2+^ metal atoms forming M–O bonds (M= Sn, Pb), while the contribution of I–H_W_ bonds between the surface iodine atoms and the water hydrogen atoms is less pronounced, Figure 2a. Fitting the tail of the g^I–Hw^(r) at low distances with a Gaussian distribution, we may estimate an average I–H_W_ bond distance of 2.73 Å for MAPbI_3_ and 2.91 Å for MASnI_3_, indicating a stronger interaction at the MAPbI_3_/water interface. Both perovskite slabs show a lack of long-range correlation between the surface atoms and the water environment. Note that, within feasible AIMD time scales, the interaction of the perovskite slabs and the water environment mainly occurs at the surface, as visible in the M/O (M = Sn, Pb) g(r) in Figure S1, Supporting Information. Despite the pronounced Sn–O bond structure, the amount of water molecules adsorbed at the perovskite surface via M–O bonds is higher for MAPbI_3_, on average 0.88 ± 0.09 adsorbed water molecules per surface Pb compared to 0.69 ± 0.05 adsorbed water molecules per surface Sn atom, Figure 2b. Previous DFT studies^48^ showed a substantial increase in the ionic contribution to the M–O and I–H_W_ bonds at the PbI_2_-terminated MAPbI_3_ surface compared to the SnI_2_-terminated MASnI_3_ surface. This should lead to an increased attraction of the water oxygen atoms at the MAPbI_3_ surface and may explain the g^I–Hw^(r) shift toward lower distances, Figure 2a, and finally in the increased number of adsorbed water molecules at the MAPbI_3_ surface.

**Figure 2:**
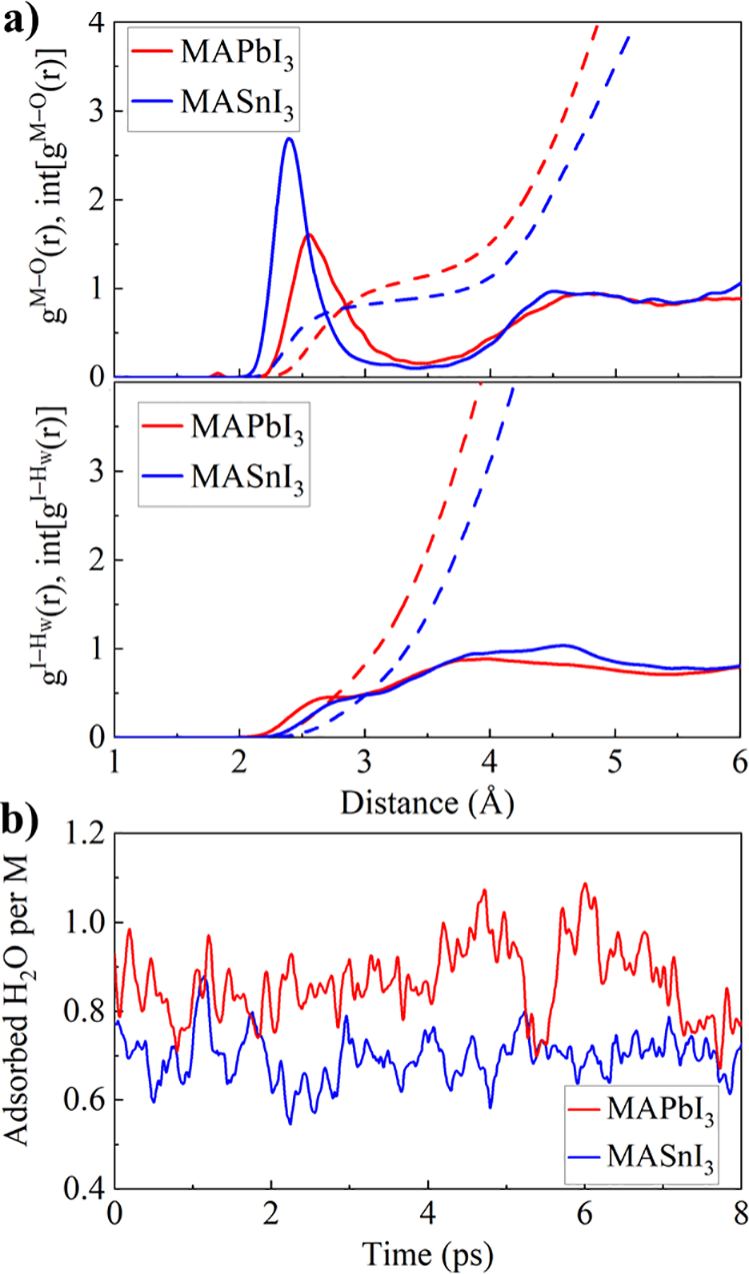
(a) M–O (M = Sn, Pb) and I–H_W_ radial pair distribution, g^M–O^(r) and g^I–Hw^(r), and the integrated distribution, int[g^M–O^(r)] and int[g^I–Hw^(r)], for MAPbI_3_ (red) and MASnI_3_ (blue). (b) Time evolution of the number of adsorbed H_2_O molecules per surface metal atom, identified by M–O bonds below 3.0 Å.

We now move on to study the structural properties at the hydrated perovskite surfaces and bulk for MAPbI_3_ and MASnI_3_. We observe substantial differences in the surface structure when considering the time average for each atom over the AIMD trajectory, Figure 3a. Surprisingly, despite the substantial interaction of the MAPbI_3_ surface atoms with the water environment, the time-averaged MAPbI_3_ surface seems only weakly distorted. In contrast, the MASnI_3_ surface shows a strong structural distortion in the inorganic Sn–I framework and indicates broken Sn–I bonds in the surface planes and in between the layers. In the time-averaged MAPbI_3_/water interface, we observe a water molecule which has entered the MAPbI_3_ bulk being located in the first MAI layer, as reported in our previous study.^26^

**Figure 3:**
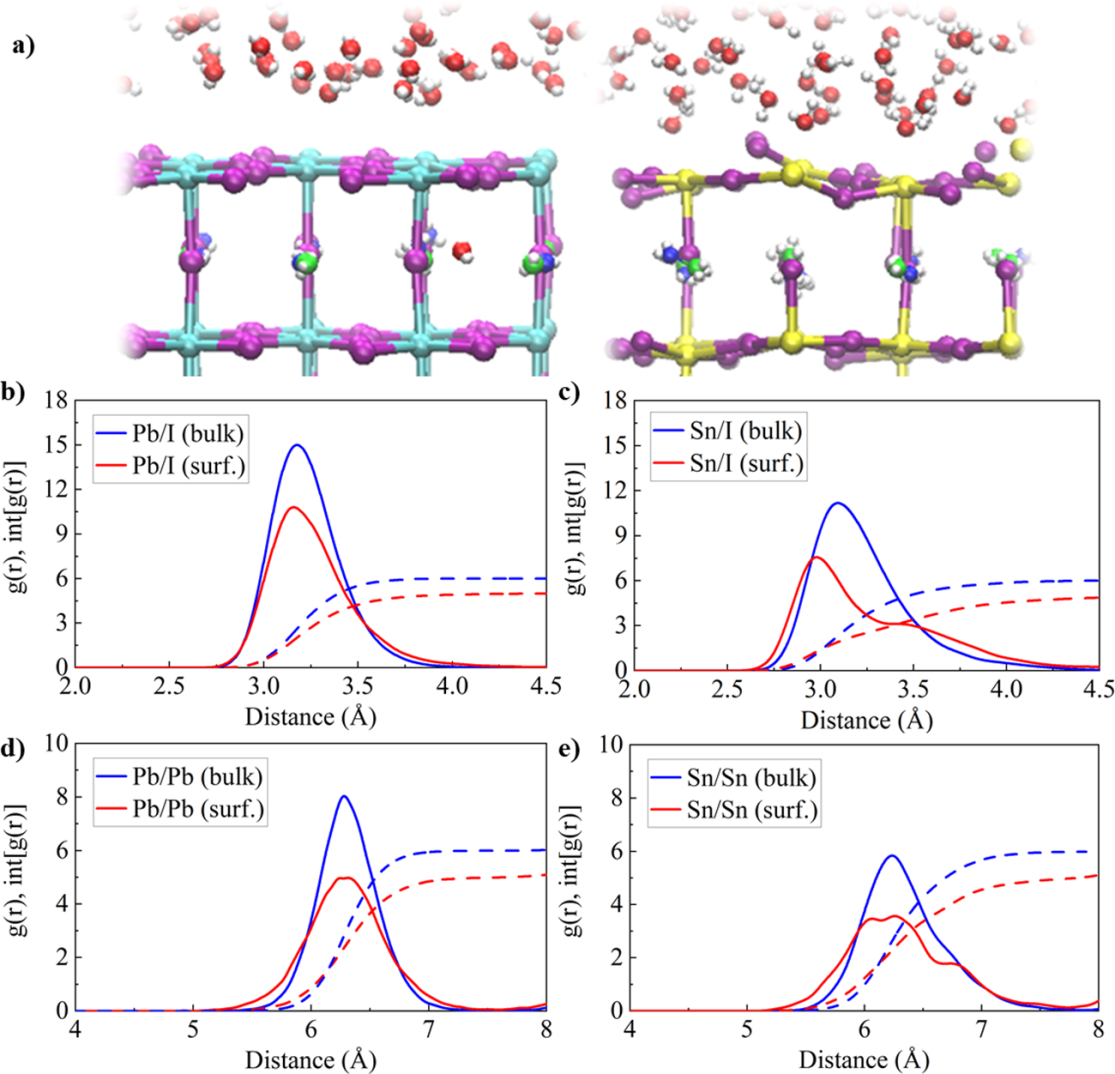
(a) Time-averaged structure for (left) MAPbI_3_/water and (right) MASnI_3_/water interface. Panels (b) and (c) give the Pb/I and Sn/I radial pair distribution, g(r) (solid), and the integrated distribution, int[g(r)] (dashed), for MAPbI_3_ and MASnI_3_, respectively. Panels (d) and (e) give the Pb/Pb and Sn/Sn g(r) (solid) and int[g(r)] (dashed) for MAPbI_3_ and MASnI_3_, respectively. In (c–e), we distinguish between the surface, defined by the outermost MI_2_ layers (M = Sn, Pb), and the bulk, defined by the inner layers. The notation, e.g., Sn/I (surf.), represents the g(r) of surface Sn atoms with all (surface and bulk) I atoms.

To quantify these visual observations, we compare the radial pair distributions for the individual components in both MAPbI_3_ and MASnI_3_. Note that we distinguish between surface atoms, given by the atoms located within the outer PbI_2_ and SnI_2_ layers, and identify the remaining atoms as bulk. The Pb/I bulk g(r), Figure 3b, shows a sharp peak at 3.17 Å, being near the experimental bond Pb–I length of ∼3.19 Å,^49^ and its integral reaches the expected coordination number of 6 within 3.8 Å. At the surface, the peak is shifted to 3.14 Å and slightly broadened; the Pb/I coordination number of surface Pb atoms reaches the expected value of 5 within 4.1 Å. The bulk and surface Pb/Pb g(r) both reach the maximum at 6.30 Å, while the surface Pb/Pb g(r) is slightly broadened, Figure 3d. Unlike for MAPbI_3_, we observe substantial differences between the surface and the bulk g(r) for MASnI_3_. The bulk Sn/I g(r), Figure 3c, is substantially broadened with a maximum at 3.07 Å, and the int[g(r)] reaches the expected Sn/I coordination number of 6 within 4.31 Å; surface Sn/I g(r) substantially differs from the bulk and shows a sharp peak at 2.99 Å, representing the equatorial Sn–I bonds, and a broadened contribution at large distances centered at around 3.48 Å, representing the axial Sn–I bonds between surface Sn and I from the first MAI layer. The Sn/I coordination number slowly increases with distance and reaches the expected value of 5 after 5.11 Å, which clearly points to elongated and potentially even broken bonds as observed in Figure 3a. The interaction of the water environment with the MASnI_3_ surface further broadens the surface Sn/Sn g(r), Figure 3e, while the bulk Sn/Sn g(r) remains at the expected Sn–Sn distance of 6.21 Å. Interestingly, the I/I g(r) do not show substantial differences between the hydrated surfaces and the bulk for both of the considered perovskites, Figure S2, Supporting Information.

It is also interesting to investigate the structural properties of the MA cations in terms of the metal/MA g(r) and the orientation of the MA within the inorganic cages. The apparent stability of the MAPbI_3_ surface is also reflected in the Pb/MA g(r), Figure S2c, Supporting Information. We observe a broad distribution for both the bulk and the surface, which, however, follow the same shape. In contrast, the Sn/MA g(r) of the surface Sn atoms varies from the bulk and shows a flattened distribution, which indicates a lack of correlation between the surface Sn atoms and the MA cations. Interestingly, the MA cations in the MAI layers near the SnI_2_-terminated surface are highly oriented at ±30° (layer 1 and layer 4 in Figure S3b, Supporting Information), while MA orientation in the hydrated MAPbI_3_ is strongly perturbed in comparison to the bare MAPbI_3_ slabs.^26^ Despite the strong structural disorder in the hydrated MASnI_3_ surface, the I/MA g(r) of surface I atoms follows the bulk g(r) closely, Figure S2f, Supporting Information, which may explain the high orientation of the MA cations in MASnI_3_.

To establish the main water-induced degradation mechanism of the MASnI_3_ surface, we now analyze the temporal evolution of the surface bonds in terms of the Sn/I coordination number in comparison to MAPbI_3_, Figure 4a. The Pb/I coordination number in the MAPbI_3_ bulk remains close to 6 throughout the trajectory; at the surface, it remains approximately 5 until 5.2 ps and then drops to ∼3.5 for a short time. This is caused by a breaking of equatorial Pb–I bonds at the hydrated surface, while the axial Pb–I bonds between the surface and the first MAI layer remain stable, Figure S4, Supporting Information. The equatorial Pb–I bonds, however, are restored after a short time period such that the coordination number approaches a value of 5 again. As shown in our previous study, the fundamental degradation mechanism of the MAPbI_3_ surface is initiated by the infiltration of the water molecule,^26^Figure 4b. Recent classical molecular dynamics simulations underlined that water infiltration into the perovskite bulk is a crucial step to initiate the degradation of PbI_2_-terminated surfaces.^32^ Within the AIMD trajectory, we did not observe the infiltration of water molecules for the MASnI_3_ surface. The Sn/I coordination number of the surface Sn decreases slowly in time from ∼4.5 after the thermalization period (first ∼2 ps) to ∼3.5 at 8 ps, Figure 4a. The origin of this change in coordination number is the breaking of axial bonds between the surface Sn atoms and the I atoms in the first MAI layer. The average Sn–I interlayer distance increases from ∼3.5 Å at 3.7 ps to a maximum of ∼4.0 Å at 6.8 ps, Figure 4c, while individual Sn–I distances above 5.4 Å can be observed, Figure 4d. In contrast, the Pb–I interlayer distance remains stable at around 3.2 Å as the main effect of the water is on the equatorial Pb–I bonds, without strongly changing the axial Pb–I bonds.

**Figure 4:**
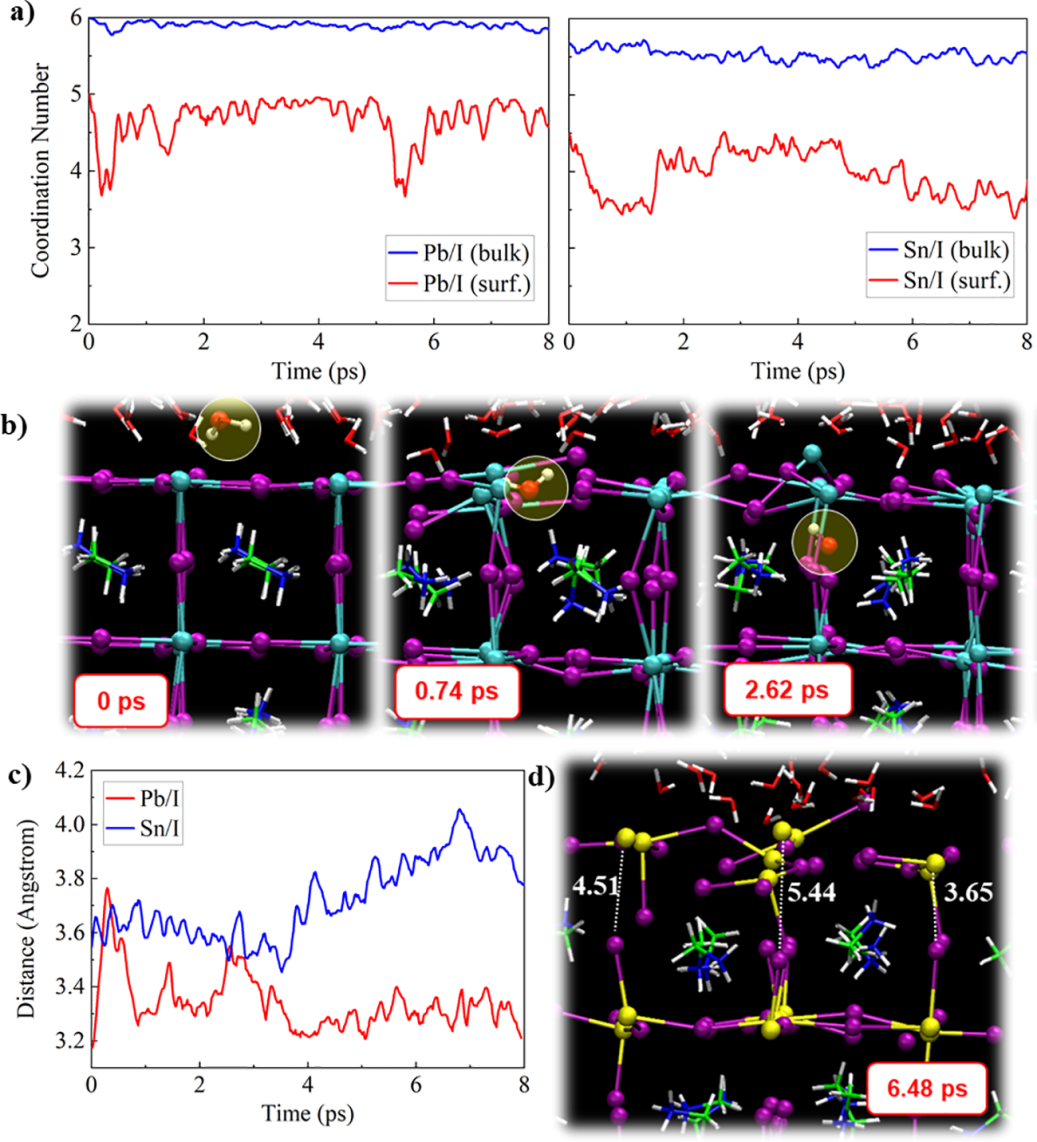
(a) Time evolution of the (left) Pb/I and (right) Sn/I coordination number in the bulk and at the surface. (b) Key steps of the infiltration of the water molecule into the MAPbI_3_ slab. (c) Time evolution of the average interlayer distance between the surface metal atoms and the iodine atoms in the first MAI layer. (d) Structure visualizing the dissolution of the MASnI_3_ surface; interlayer distances are given for several Sn–I pairs, in units of Angstrom.

The presented observations give clear indication of a *facile solvation of the SnI*_2_*-terminated surface by water*. As a consequence, we may expect an enhanced surface defect density in humid conditions which can introduce deep trap states and strongly suppresses the device performance of THP solar cells.^21^ On a longer time scale, water may easily enter the MASnI_3_ bulk through the dissolved surface to accelerate the degradation^32^ and hamper the electronic properties.^42^ This emphasizes the need for perovskite synthesis under a controlled moisture-free environment and additionally may require passivation of MASnI_3_ using hydrophobic protection layers. Note that we previously observed the rapid solvation of the MAI-terminated MAPbI_3_ surface from AIMD simulations, which occurs by the release of I atoms under the attack of water molecules at Pb sites.^26^ Further DFT studies reported comparable water adsorption energies on MAI-terminated MAPbI_3_ and MASnI_3_ surfaces.^48^ We thus are confident that the degradation process of the MAI-terminated MASnI_3_ will follow a similar mechanism as in MAPbI_3_.

We now analyze the results on the (001) SnBr_2_-terminated orthorhombic DMASnBr_3_/water interface to gain a deeper understanding of the origin of the high water stability of DMASnBr_3_.^46,50^ The g^Sn–O^(r) of the DMASnBr_3_ surface shows a maximum at 2.37 Å, close to the Sn–O peak of MASnI_3_ (2.43 Å), while the g^Br–Hw^(r) is substantially shifted with respect to MASnI_3_ to shorter distances, Figure 5a. Additionally, the intensity of the DMASnBr_3_ g^Sn–O^(r) is less pronounced, which we may attribute to Sn–Br bonds being stronger than Sn–I ones. The overall amount of adsorbed water molecules per surface Sn atom does not substantially vary between the considered THPs, Figure S5, Supporting Information. Furthermore, the DMASnBr_3_ int[g(r)] follows closely the one of MASnI_3_, suggesting a comparable interaction of the perovskite surface and water environment. In contrast to the substantial distortion of the Sn–I bonds at the MASnI_3_ surface, however, the Sn/Br g(r) indicates highly stable metal-halide bonds at the DMASnBr_3_ surface, Figure 5b. Interestingly, the short-range correlation, given by the first peak in the Sn/Br g(r), is nicely preserved, but the second peak is flattened, which points to a rather disordered surface structure. Also, the Br/Br g(r) is preserved at the surfaces, Figure S6, Supporting Information. The Sn/Br coordination number does not strongly change with time, with an average of 3.61 and 2.99 of the bulk and of the surface Sn atoms, respectively, Figure S7, Supporting Information. In contrast to MASnI_3_, the interlayer distance between the surface Sn atoms and the Br atoms in the first DMABr layer remains relatively stable but shows larger fluctuations, Figure S8, Supporting Information. The most significant difference to MASnI_3_ is the highly disordered Sn/Sn g(r) at the DMASnBr_3_ surface, Figure 5c. While the bulk Sn/Sn distribution shows a well-pronounced peak at 5.97 Å, the surface Sn/Sn g(r) lacks both short- and long-range order. Such a distribution is atypical for a crystalline solid and points to a rather amorphous phase.

**Figure 5:**
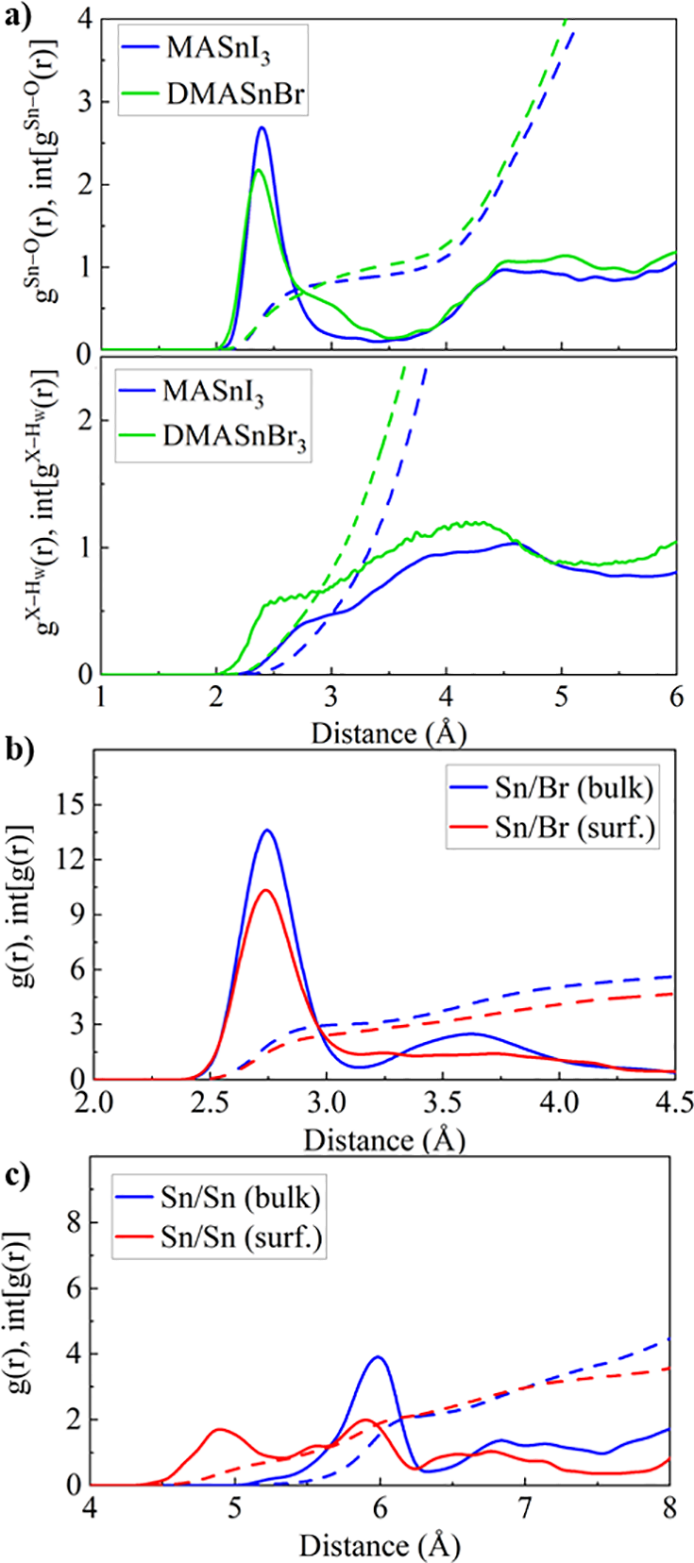
(a) Sn–O and X–H_W_ (X = Br, I) radial pair distribution and the integrated distribution for MASnI_3_ (blue) and DMASnBr_3_ (green). (b) Sn/Br and (c) Sn/Sn g(r) of DMASnBr_3_ in the bulk (blue) and at the surface (red).

Further inspection reveals, indeed, that the DMASnBr_3_ surface consists of disconnected, *zero-dimensional SnBr*_3_*complexes which form a surface layer possibly protecting the inner bulk structure*. Surface Sn atoms form three stable Sn–Br bonds with well-preserved Sn/Br and Br/Br g(r) and lack of Sn/Sn correlations, which are strong signatures of such 0D-SnBr_3_ complexes, Figure S9, Supporting Information. A comparison of the crystal structures of DMASnBr_3_ and MASnBr_3_ (Figure S10, Supporting Information) shows equal Sn–Br bond lengths along the (001) direction for MASnBr_3_ and alternating short (∼2.74 Å) and long Sn–Br bonds (∼3.45 Å) for DMASnBr_3_. Consequently, the presence of SnBr_3_ complexes within DMASnBr_3_ can be attributed to the large DMA cation. Recent AIMD simulations also observed the presence of partially decoupled SnBr_3_ complexes within formamidinium tin bromide, FASnBr_3_,^51^ which form instantaneously caused by the large size of the FA cation. Water molecules form strong Sn–O bonds with such surface complexes, which is supported by DFT calculations of the water adsorption energy, with *E*_ads_(DMASnBr_3_) = −0.72 eV to *E*_ads_(MASnI_3_) = −0.55 eV, Figure S11, Supporting Information. Relaxing the DMASnBr_3_ slab at *t* = 8 ps after removal of the water molecules does not lead to a more stable structure, Figure S12, Supporting Information, which indicates that the amorphous surface layer is formed upon interaction with water molecules at finite temperatures. Within the simulated time scales, water molecules neither enter into the perovskite nor are able to dissolve the surface. The 0D-SnBr_3_ complexes can easily rearrange at the surface to bond with water molecules and consequently to prohibit water from entering the surface. Moreover, the water environment cannot easily break the strong Sn–Br bonds, which limits the solubility of the DMASnBr_3_ surface by water. As a final remark, DMASnBr_3_ is further stabilized by the relatively strong hydrophobic nature of the DMA cation with respect to MA.^46^ As a consequence, higher stability of DMABr-terminated surfaces compared to MASnI_3_ may be expected due a reduced solvation of DMA cations, and water infiltration into the SnBr_2_-terminated DMASnBr_3_ surface may be further suppressed. The high band gap (∼2.8 eV^50^) in combination with the amorphous surface layer may prevent the applicability of DMASnBr_3_ in photovoltaics. On the contrary, this inherent surface stability can be the crucial aspect for exploitation of DMASnBr_3_ as water-stable THP in photocatalytic applications.

In summary, we have reported *ab initio* molecular dynamics simulations to investigate the surface degradation of tin-halide perovskites using two prototypical systems, i.e., MASnI_3_ and DMASnBr_3_, and provided a comparison with the prototypical lead-based counterpart MAPbI_3_. Our results show a facile dissolution of the SnI_2_-terminated MASnI_3_ surface under water. Water molecules bond with the surface Sn atoms and induce the breaking of axial Sn–I bonds with the I atoms of the adjacent MAI layer. Our AIMD results predict a higher water stability of the PbI_2_-terminated MAPbI_3_ surface. Despite showing an increased number of adsorbed water molecules on the MAPbI_3_ surface, the lead iodine bonds remain stable throughout the trajectory without showing any substantial difference in the radial pair distribution between the bulk and the surface. Still, water molecules may enter the MAPbI_3_ bulk where they may initiate the solvation of the perovskite.^26,32^ Finally, we reported AIMD simulations to analyze the origin of the experimentally reported^46,50^ surface stability of DMASnBr_3_ in a liquid water environment. We observed highly stable Sn–Br bonds at the surface and an increased stability of the inorganic framework along the *c*-axis. Our results show that the DMASnBr_3_ surface is made of disconnected, zero-dimensional SnBr_3_ complexes which form an amorphous layer that can easily reorient within the surface and hereby protect the DMASnBr_3_ bulk. Such amorphous surface structures typically are believed to be detrimental in terms of photovoltaics but may be key for the applicability of THPs in photocatalysis. Overall, we believe that the reported atomistic details of the THP/water interface in this work will inspire novel surface modification and device engineering strategies to enhance the stability of THPs for lead-free perovskite photovoltaics and photocatalysis.

**Computational Details.** The models of the THP–water interfaces are constructed by five SnX_2_ (X = I, Br) layers such that the perovskite slabs are terminated by SnX_2_ layers, Figure 1. The cell parameters in the *a* and *b* axes are fixed to the experimental values: MASnI_3_*a* = *b* = 17.5154 Å,^52^ DMASnBr_3_*a* = 12.274 Å, *b* = 12.071 Å,^46^ representing 2 × 2 (001) slabs of the tetragonal MASnI_3_ and of the orthorhombic DMASnBr_3_ phases. A vacuum region of 15 Å was added on top of the perovskite slabs, such that MASnI_3_*c* = 47.709 Å and DMASnBr_3_*c* = 47.5 Å. The vacuum region was filled by 155 and 72 water molecules for the MASnI_3_ and the DMASnBr_3_ model, respectively, thus giving rise to a liquid water environment characterized by the experimental density of liquid water. We reproduce part of our previous AIMD analysis on the water-induced degradation of MAPbI_3_,^26^ together with additional statistical analysis, to provide a direct comparison with the prototypical MAPbI_3_/water interface as representative of the lead-based counterpart.

We carried out AIMD simulations on the THP–water interface models using the Quickstep module in the CP2K software package.^53−56^ We used a double-ζ basis set (DZVP-MOLOPT)^57^ combined with the norm-conserving Goedecker–Teter–Hutter (GTH) pseudopotentials^58^ and a CUTOFF = 800 Ry, and REL_CUTOFF = 50 Ry for the expansion of the electron density. The revised PBE (revPBE) parametrization of the PBE functional was used to account for the exchange and correlation potentials. van der Waals corrections are included by means of the rVV10 functional to account for nonlocal electron correlations.^59^ The temperature was controlled by a Nosé–Hoover thermostat^60,61^ with a target temperature of 350 K and a time constant of 16.68 fs, and the volume was kept constant by applying an NVT ensemble. The integration time step of the dynamics was set to 0.48 fs. The AIMD simulations were run for at least 8 ps for both the MASnI_3_/water and the DMASnBr_3_/water interface model.

## Supporting Information Available

Radial pair distribution functions for MAPbI_3_ and MASnI_3_; methylammonium orientation in the individual MAI layers; snapshot visualizing the equatorial Pb–I bond breaking at the hydrated MAPbI_3_ surface; time evolution of the adsorbed water molecules at the MASnI_3_ and the DMASnBr_3_ surface; Sn/DMA and Br/Br radial pair distribution functions for DMASnBr_3_; AIMD snapshot of the DMASnBr_3_–water interface visualizing SnBr_3_ complexes; crystal structures of MASnI_3_, MASnBr_3_, and DMASnBr_3_; DFT calculations of the adsorption energy of a single water molecule on the perovskite surfaces; optimized DMASnBr_3_ slabs before and after the interaction with water (PDF)
