# Supplementary material for: Stability of Tin- versus Lead-Halide Perovskites: Ab Initio Molecular Dynamics Simulations of Perovskite/Water Interfaces
Source: J Phys Chem Lett. 2022 Mar 4;13(10):2321–9. doi: 10.1021/acs.jpclett.2c00273 (PMC8935372; doi:10.1021/acs.jpclett.2c00273)
Supplement: Supplementary file 1 — jz2c00273_si_001.pdf [file jz2c00273_si_001.pdf]

Supporting Information

Stability of Tin- *vs*. Lead-Halide Perovskites: *Ab Initio* Molecular Dynamics Simulations of Perovskite/Water Interfaces

Waldemar Kaiser,[1*] Damiano Ricciarelli,[1,2] Edoardo Mosconi,[1,3] Asma A. Alothman,[3] Francesco Ambrosio,[1,4,5*] Filippo De Angelis[1,2]

[1]*Computational Laboratory for Hybrid/Organic Photovoltaics (CLHYO), Istituto CNR di Scienze e Tecnologie Chimiche "Giulio Natta" (CNR-SCITEC), Via Elce di Sotto 8, 06123 Perugia, Italy.*

[2]*Department of Chemistry, Biology and Biotechnology, University of Perugia, Via Elce di Sotto 8, 06123 Perugia, Italy.*

[3]*Chemistry Department, College of Science, King Saud University, Riyadh 11451, Kingdom of Saudi Arabia.*

[4] *Department of Chemistry and Biology "A. Zambelli", University of Salerno, Via Giovanni Paolo II 132, 84084 Fisciano, Salerno, Italy.*

[5] *CNST@Polimi, Istituto Italiano di Tecnologia, Via Pascoli 70/3, 20133 Milano, Italy.*

Corresponding Author

E-Mail: waldemar.kaiser@scitec.cnr.it ; fambrosio@unisa.it

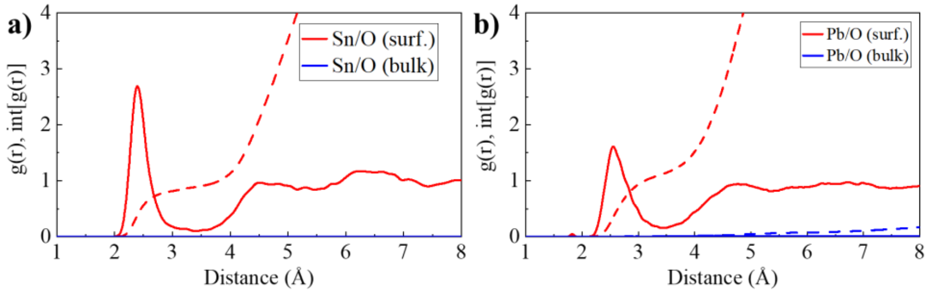

Figure S1. (a) Time-averaged Sn/O radial pair distribution function, g(r), and its integral, int[g(r)], at the surface (red) and in the bulk (blue) for $MASnI_3$. (b) Time-averaged Pb/O g(r) and int[g(r)] at the surface (red) and in the bulk (blue) for $MAPbI_3$.

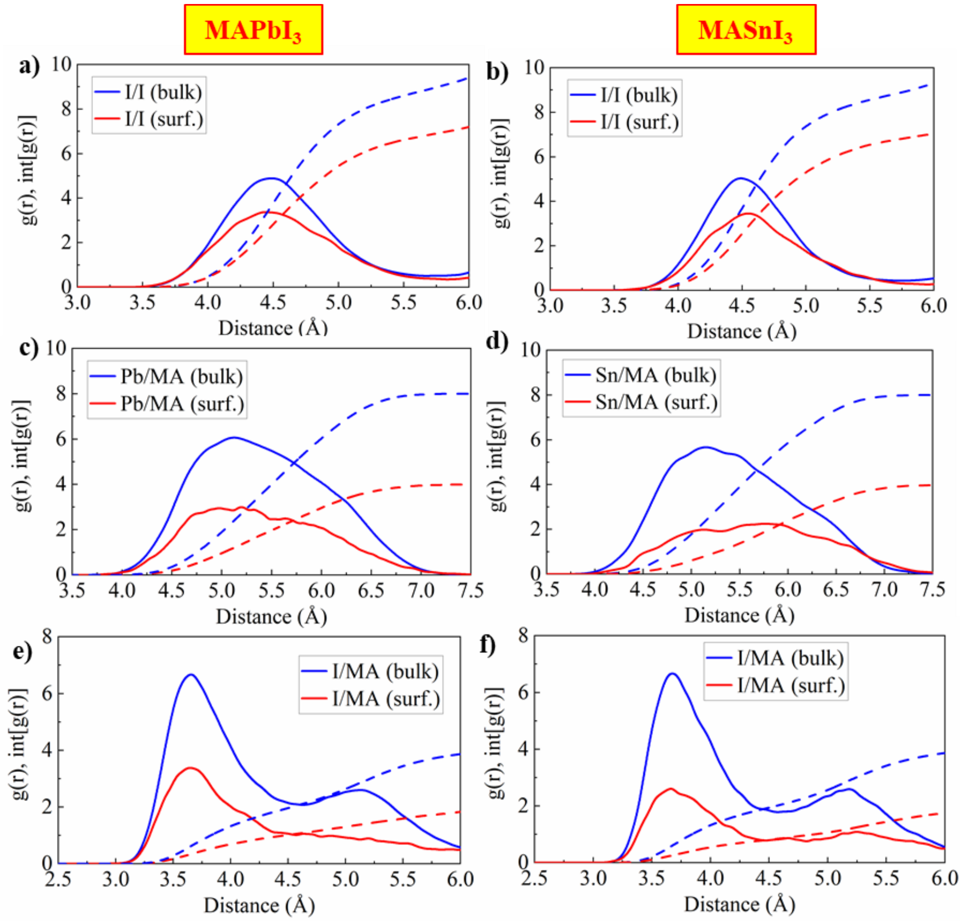

Figure S2. (a, b) I/I, (c, d) M/MA (M = Sn, Pb, MA = methylammonium), and (e, f) I/MA radial pair distribution functions, g(r) (solid lines), and integrated radial pair distribution functions, int[g(r)] (dashed lines), for $MAPbI_3$ and $MASnI_3$.

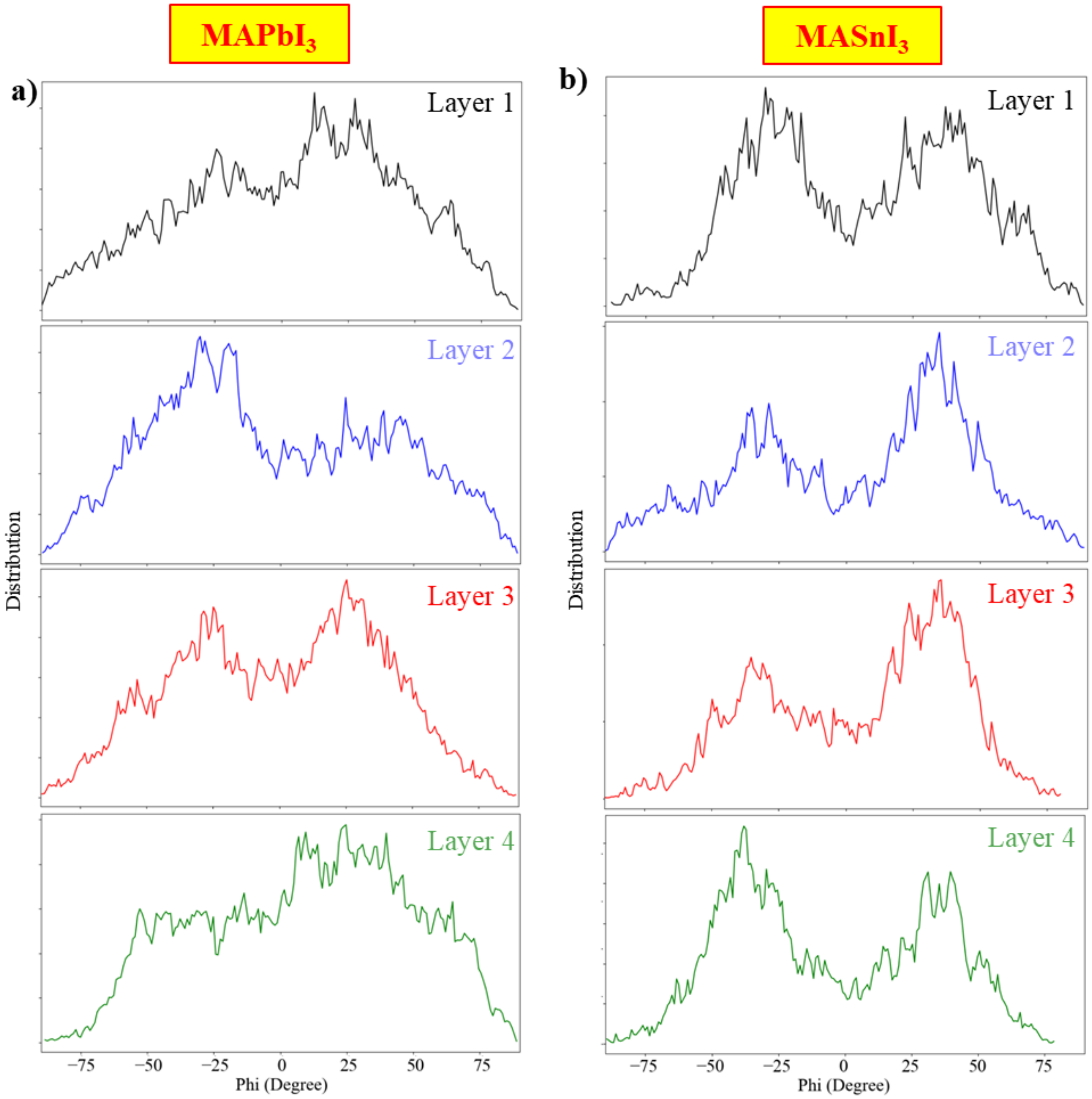

Figure S3. Distribution of the angles formed between the MA C-N axis and the perovskite *ab*-plane for the hydrated (a) $MAPbI_3$ and (b) $MASnI_3$ perovskite slabs separated for each MAI layer.

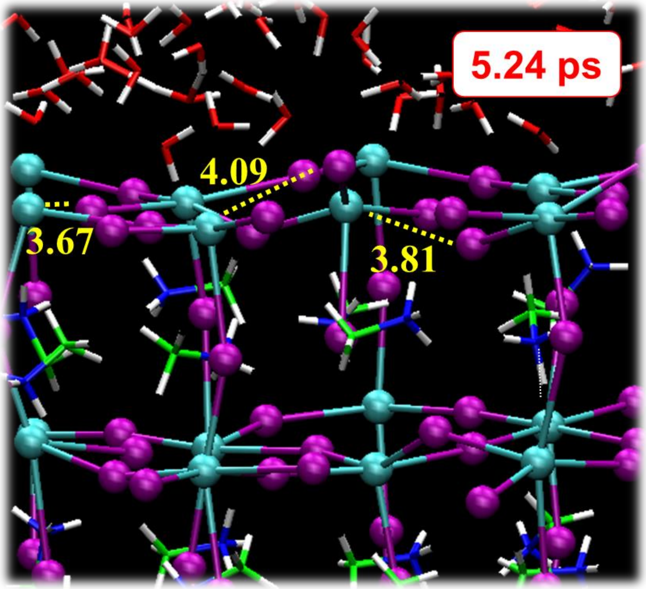

Figure S4. Snapshot of the hydrated $MAPbI_3$ surface at 5.24 ps. Broken Pb–I bonds are highlighted by dashed lines, distances are given for several Pb–I pairs, in units of Angstrom.

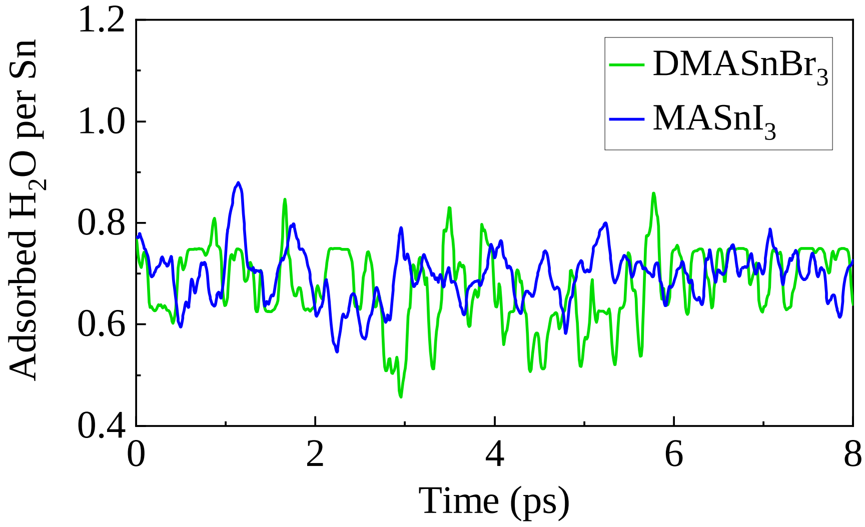

Figure S5. Time evolution of the number of adsorbed H_2O molecules per surface Sn atom for $DMASnBr_3$ and $MASnI_3$, identified by Sn–O bonds below 3.0 Å.

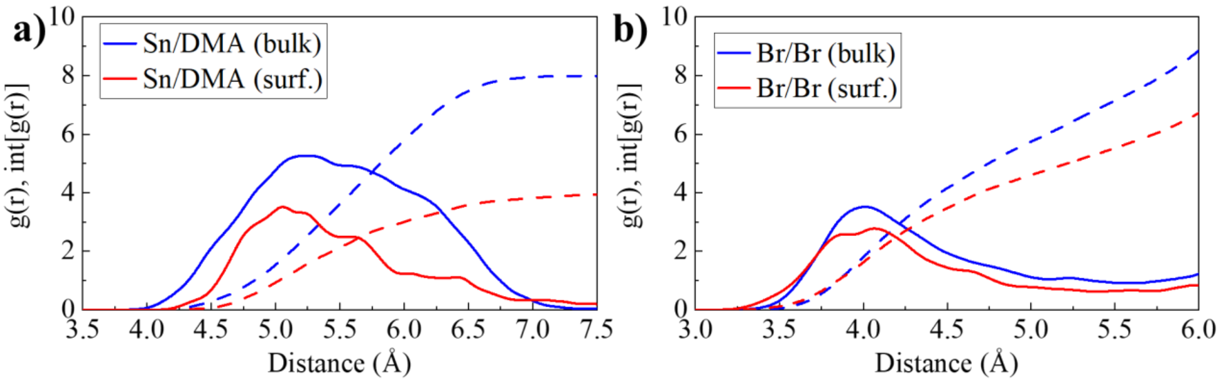

Figure S6. (a) Sn/DMA and (b) Br/Br radial pair distribution and the integrated distribution for $DMASnBr_3$.

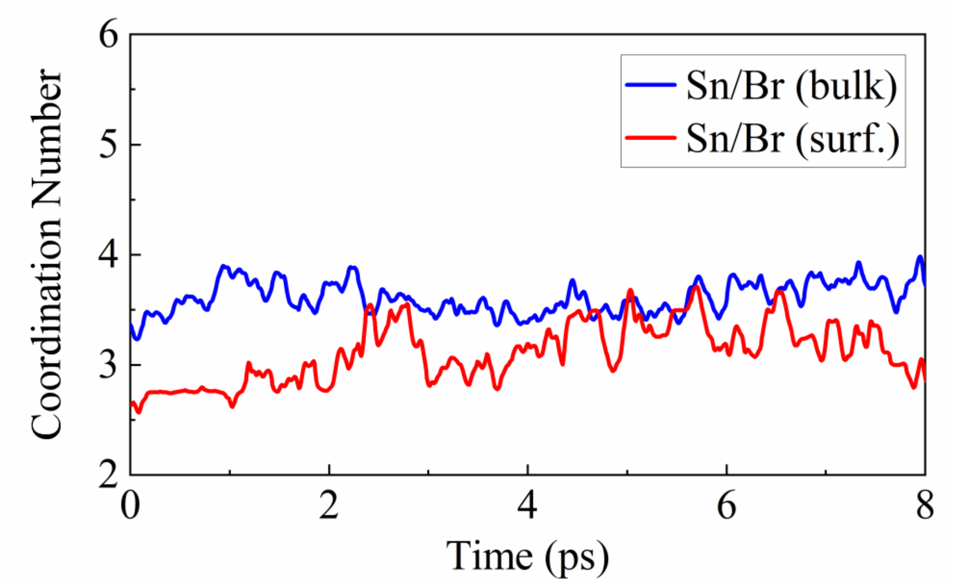

Figure S7. Time evolution of the Sn/Br coordination number at the surface (red) and in the bulk (blue) for $DMASnBr_3$.

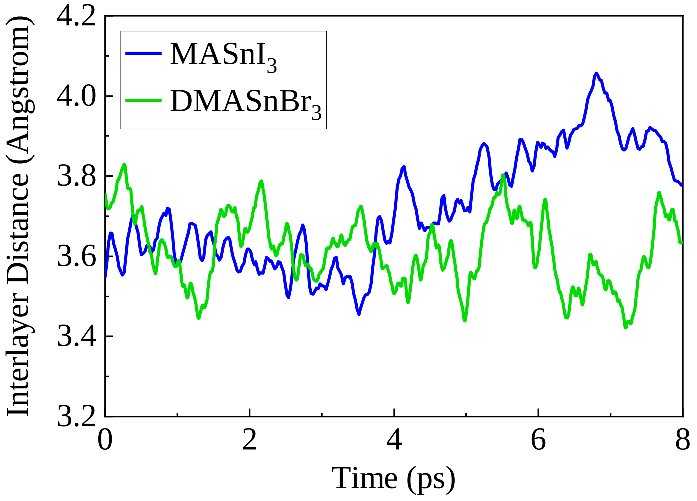

Figure S8. Time evolution of the average interlayer distance between the surface Sn atoms and the bromine atoms in the first DMABr and MAI layer for $DMASnBr_3$ and $MASnI_3$, respectively.

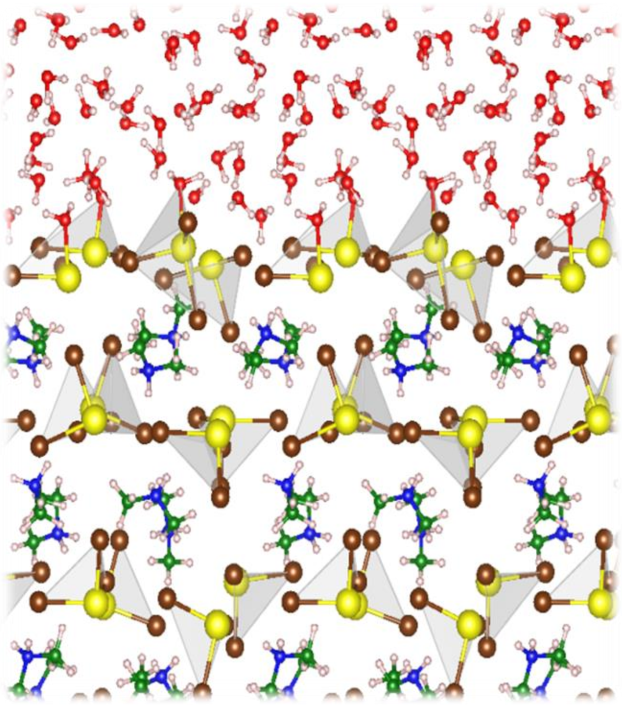

Figure S9. Snapshot of the $DMASnBr_3$-water interface at t=8 ps of AIMD simulation time. Polyhedra between the tin, bromine, and water oxygen atoms are visualized to highlight the hydrated zero-dimensional $SnBr_3$ complexes at the surface.

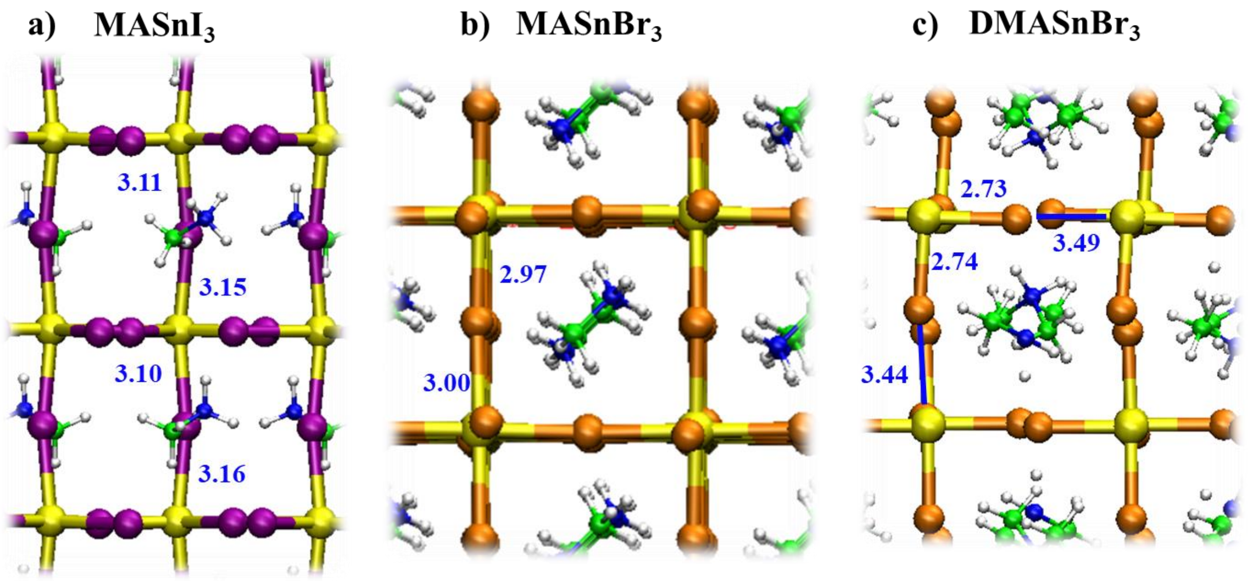

Figure S10. Crystal structure of (a) $MASnI_3$, (b) $MASnBr_3$, and (c) $DMASnBr_3$. In each structure the Sn-X (X=I, Br) bond lengths along the (001) direction are given in units of Å.

Computational details of the water adsorption energy calculation

Geometry optimization of the perovskite slabs with adsorbed water molecule have been carried out using the Quantum Espresso PWscf code[1] using the GGA-PBE functional,[2] with plane-wave basis set cutoffs for the smooth part of the wave functions and the augmented density of 40 and 320 Ry, respectively. For all calculations, electron−ion interactions were described by scalar relativistic ultrasoft pseudopotentials with electrons from O, N, and C 2s, 2p; H 1s; I 5s, 5p; Br 4s, 4p; Sn 5s, 5p, 4d; and Pb 6s, 6p, 5d shells explicitly included in the calculations.

The adsorption energy is calculated as $\Delta E(ads) = E(slab + water) - E(water) - E(slab)$ and visualized in Figure S10.

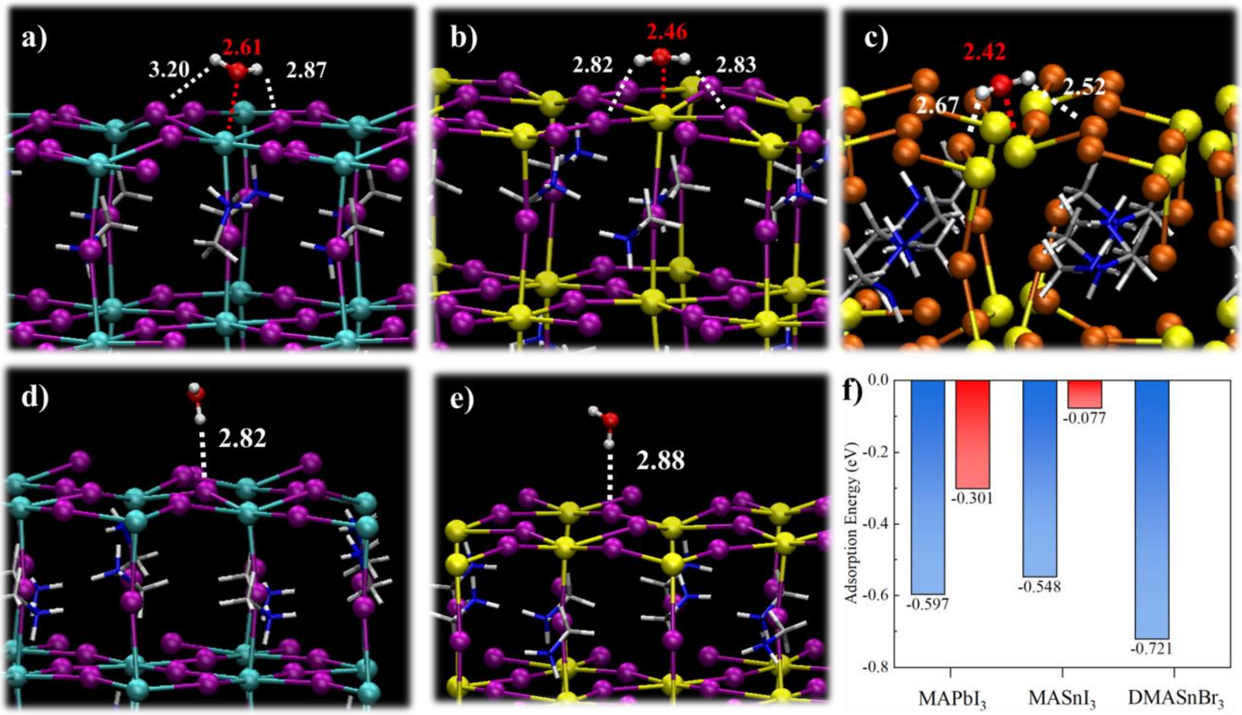

Figure S11. Adsorption of a single water molecule on perovskite surfaces. (a), (b) and (c) visualize the optimized structures of the adsorbed water molecule on $MAPbI_3$, $MASnI_3$, and $DMASnBr_3$, respectively, by formation a bond between a surface metal atom (Sn, Pb) and the water oxygen atom. (d) and (e) visualize the optimized structures of the adsorbed H_2O molecule on $MAPbI_3$ and $MASnI_3$, respectively, by formation of a I–H_w bond; all attempts to form a Br–H_w bond resulted in the formation of a Sn–O bond. Dashed lines visualize the respective bonds; the adjacent numbers give the bond distance in units of Å. (f) Adsorption energies of the water molecule on the different perovskites: blue bars represent the scenarios (a) to (c), red bars represent scenarios (d) and (e).

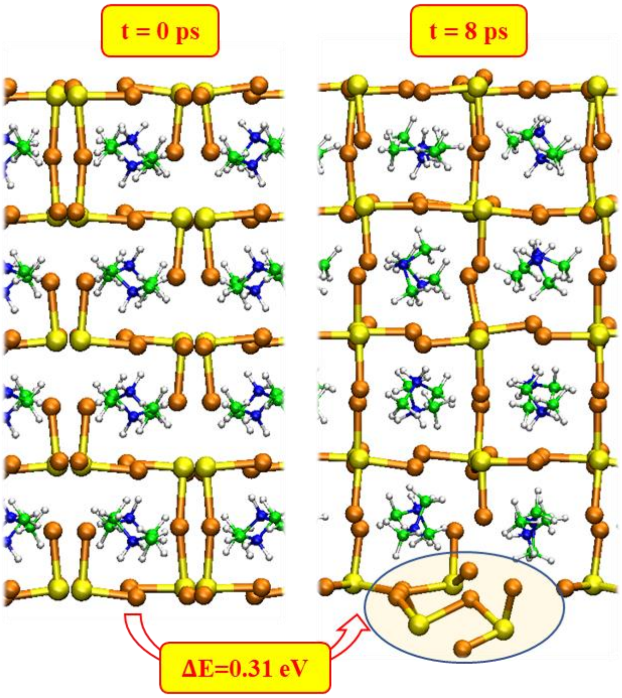

Figure S12. (a) Optimized structures of the $DMASnBr_3$ slabs taken at t=0 ps and t=8 ps upon removal of the water molecules. The optimized geometry at 8 ps shows a Sn_2Br_6 complex at the surface as highlighted. The initial $DMASnBr_3$ perovskite is of 0.31 eV more favorable compared to the 8 ps structure, which highlights that the 8 ps geometry is not stable.

References

(1) Giannozzi, P.; Baroni, S.; Bonini, N.; Calandra, M.; Car, R.; Cavazzoni, C.; Ceresoli, D.; Chiarotti, G. L.; Cococcioni, M.; Dabo, I.; Dal Corso, A.; Gironcoli, S. de; Fabris, S.; Fratesi, G.; Gebauer, R.; Gerstmann, U.; Gougoussis, C.; Kokalj, A.; Lazzeri, M.; Martin-Samos, L.; Marzari, N.; Mauri, F.; Mazzarello, R.; Paolini, S.; Pasquarello, A.; Paulatto, L.; Sbraccia, C.; Scandolo, S.; Sclauzero, G.; Seitsonen, A. P.; Smogunov, A.; Umari, P.; Wentzcovitch, R. M. QUANTUM ESPRESSO: A Modular and Open-Source Software Project for Quantum Simulations of Materials. *J. Phys. Condens. Matter* **2009**, *21* (39), 395502. DOI: 10.1088/0953-8984/21/39/395502.

(2) Perdew, J. P.; Burke, K.; Ernzerhof, M. Generalized Gradient Approximation Made Simple. *Phys. Rev. Lett.* **1996**, *77* (18), 3865–3868. DOI: 10.1103/PhysRevLett.77.3865.
